# Comparison of the Type, Impact, and Resolution of Complications Following Direct Anterior Versus Posterior Approach Total Hip Arthroplasty: A Multicenter Propensity-Weighted Analysis

**DOI:** 10.2106/JBJS.OA.26.00172

**Published:** 2026-07-17

**Authors:** Sergio F. Guarin Perez, Heather J. Roberts, Diego J. Restrepo, Abraham Babalola, Dirk R. Larson, Cameron K. Ledford, Joshua S. Bingham, Mark W. Pagnano, Robert T. Trousdale, Michael J. Taunton, Rafael J. Sierra

**Affiliations:** 1 Department of Orthopedic Surgery, Mayo Clinic, Rochester, Minnesota; 2 Department of Quantitative Health Sciences, Mayo Clinic, Rochester, Minnesota; 3 Department of Orthopedic Surgery, Mayo Clinic Florida, Jacksonville, Florida; 4 Department of Orthopedic Surgery, Mayo Clinic Arizona, Phoenix, Arizona

## Abstract

**Background:**

Prior studies have reported higher rates of periprosthetic fracture, superficial infection, and neurologic deficit with the direct anterior approach (DAA), and higher rates of dislocation with the posterior approach (PA) for total hip arthroplasty (THA). Despite these differences, the overall clinical burden of complications between approaches remains unclear. The purpose of this study was to compare the profiles, severity, impact, and resolution of 5 clinically important complications between DAA and PA THA.

**Methods:**

A retrospective review was conducted of 11,783 consecutive primary THAs performed between 2010 and 2021 across 3 centers within a single healthcare system, using either the DAA (n = 4,451) or the PA (n = 7,332). Complications assessed included dislocation, infection, periprosthetic fracture (PPFx), aseptic loosening, and neurologic deficit. Severity was graded using the Clavien-Dindo classification (I = minor, V = death). Impact was defined by reoperation burden, and outcomes were categorized as resolved or persistent. Kaplan-Meier survivorship and propensity-weighted Cox proportional hazards models were used to compare complication risk.

**Results:**

The overall 1-year risk of surgical complications did not differ between DAA and PA (hazard ratio [HR], 0.89; 95% confidence interval [CI], 0.75-1.06; p = 0.208). Dislocation occurred more frequently with PA THA than DAA THA (1.9% vs. 0.4%; HR, 5.20; 95% CI, 3.04-8.90; p < 0.001). Superficial infections were more frequent with the DAA (1.6% vs. 0.8%; HR, 2.43; 95% CI, 1.68-3.53; p < 0.001). The risk of deep infection, PPFx, aseptic loosening, and neurologic deficit did not differ significantly between approaches. Among patients with complications, the proportion classified as severe (Clavien-Dindo III–V) was similar, and adjusted analyses showed no difference in the odds of severe complications (adjusted odds ratio 1.34 for PA vs DAA; 95% CI, 0.95-1.91; p = 0.099). Complication impact and resolution were likewise comparable.

**Conclusion:**

Although the profile of complications differed between DAA and PA THA, the severity, early clinical impact, and resolution of complications appeared similar. Notably, DAA was associated with a lower risk of dislocation and a higher risk of superficial infection compared with PA THA.

**Level of Evidence:**

Level III, Retrospective Comparative Study. See Instructions for Authors for a complete description of levels of evidence.

## Introduction

The direct anterior approach (DAA) has gained popularity due to its lower risk of postoperative dislocation and reported quicker recovery compared with traditional approaches in total hip arthroplasty (THA)^1,2^. Higher rates of dislocation have been reported after the posterior approach (PA)^3-6^, while the DAA has been linked to greater risks of periprosthetic fracture (PPFx), superficial infection, neurologic injury, and aseptic loosening^7-10^. Because surgical approach selection in clinical practice is influenced by both patient characteristics and surgeon preference, observational comparisons are susceptible to treatment-selection bias, with some surgeons favoring the PA for patients at higher risk of wound complications and others preferring the DAA due to concerns about dislocation^11,12^.

Although the profile of complications associated with each approach has been described, it remains unclear whether the overall clinical burden (incidence, severity, need for additional interventions, and likelihood of resolution) differs between approaches. Therefore, the purpose of this study was to compare these dimensions between DAA and PA THA.

## Materials and Methods

Institutional Review Board approval was obtained. A retrospective review of 11,783 consecutive primary THAs was conducted between 2010 and 2021. Exclusions included neoplastic disease, pathologic fracture, prior native hip infection, neuromuscular conditions, and skeletal dysplasias. There were 4,451 cases (38%) performed using the DAA and 7,332 cases (62%) performed using the PA. The mean age was 63.6 ± 13.3 years, the mean body mass index (BMI) was 30.0 ± 6.2 kg/m^2^, and 52.7% of the cohort were female. Baseline demographic and clinical characteristics differed between the DAA and the PA groups and are shown in Table I. Forty-two surgeons contributed cases to this multicenter cohort. The majority (28 of 42, 66.7%) were adult reconstruction fellowship-trained, with additional contributions from surgeons fellowship-trained in other specialties. Among the 25 surgeons contributing at least 10 cases (99.6% of the cohort), 9 performed only the PA, 1 performed only the DAA, and 15 performed both approaches. The median follow-up for the full cohort was 2.4 years (mean 3.5 years, up to 12.6 years).

**TABLE I:** Baseline Patient and Surgical Characteristics by Surgical Approach

		Approach		
	Total	Direct Anterior	Posterior	
	(N = 11,783)	(N = 4,451)	(N = 7,332)	p
Age at surgery				<0.0001*
Mean (SD)	63.6 (13.25)	64.7 (11.51)	62.9 (14.16)	
Range	18.0, 98.0	19.0, 97.0	18.0, 98.0	
Gender, n (%)				0.33562
Female	6,215 (52.7)	2,373 (53.3)	3,842 (52.4)	
Male	5,568 (47.3)	2,078 (46.7)	3,490 (47.6)	
BMI				<0.0001*
Mean (SD)	30.0 (6.23)	29.0 (5.62)	30.6 (6.49)	
Range	13.8, 68.6	15.2, 62.5	13.8, 68.6	
ASA Score, n (%)				<0.0001†
ASA = 1/2	8,008 (68.0)	3,132 (70.4)	4,876 (66.5)	
ASA = 3/4	3,775 (32.0)	1,319 (29.6)	2,456 (33.5)	
Indication, n (%)				<0.0001†
Osteoarthritis	8,837 (75.0)	3,634 (81.6%)	5,203 (70.9%)	
Dysplasia	1,040 (8.8)	212 (4.8)	828 (11.3)	
Osteonecrosis	727 (6.2)	263 (5.9)	464 (6.3)	
Posttraumatic	563 (4.8)	129 (2.9)	434 (5.9)	
Inflammatory	378 (3.2)	132 (3.0)	246 (3.4)	
Other	238 (2.0)	81 (1.8)	157 (2.1)	
Femoral head size, n (%)				<0.0001†
22	13 (0.1)	0 (0.0)	13 (0.2)	
28	652 (5.5)	102 (2.3)	550 (7.5)	
32	1,956 (16.6)	863 (19.4)	1,093 (14.9)	
36	7,941 (67.4)	3,388 (76.1)	4,553 (62.1)	
40	1,060 (9.0)	95 (2.1)	965 (13.2)	
44	157 (1.3)	3 (0.1)	154 (2.1)	
51	2 (0.0)	0 (0.0%)	2 (0.0%)	
53	1 (0.0)	0 (0.0)	1 (0.0)	
55	1 (0.0)	0 (0.0)	1 (0.0)	
Femoral stem cemented, n (%)				<0.0001†
No	10,832 (91.9)	4,330 (97.3)	6,502 (88.7)	
Yes	951 (8.1)	121 (2.7)	830 (11.3)	

* Unequal variance 2 sample *t*-test.

† χ^2^ p-value.

This study focused on complications occurring within the first postoperative year, as these events are more likely to be directly related to the procedure and perioperative period. Five specific complications were assessed: dislocation, infection, PPFx, aseptic loosening (each within 1 year of the index surgery), and neurologic deficit (within 3 months of the index surgery). Neurologic complications were defined as clinically documented postoperative neurologic deficits identified through registry data and confirmed by chart review. Severity of complications was graded according to the Clavien-Dindo classification^13^ (Supplementary Table 1), a validated system that stratifies surgical complications based on the interventions required to manage them and is widely used as an objective measure of complication severity^14,15^. Complication impact was defined as the number of additional interventions required (reoperations, closed reductions, or revision arthroplasties), assessed at each patient's latest clinical or radiographic follow-up. Definitions of resolution for each complication type are provided in Supplementary Table 1. As patients may experience more than 1 complication, the raw complication rate reflects all events, while Kaplan-Meier survival estimates reflect time to the first (index) complication only. Complications were initially identified through the institutional arthroplasty registry. Each event was then verified through targeted review of the electronic medical record and radiographic studies to confirm the diagnosis, timing, management, and clinical outcome. Data abstraction was performed by 3 independent reviewers, each responsible for a defined portion of the cohort, with oversight to ensure consistent complication classification and the Clavien-Dindo grading.

### Statistical Analysis

The results are reported descriptively as mean (standard deviation) for continuous variables and count (percentage) for categorical variables, unless otherwise noted. Complications were analyzed as time-to-event outcomes using survivorship methodology, including Kaplan-Meier estimation and Cox regression. To balance the 2 surgical approach groups across important demographic and baseline factors, propensity score weighting was used. The propensity score was estimated using a logistic regression model with surgical approach (DAA vs. PA) as the dependent variable with the following covariates: age at surgery, sex, BMI, ASA physical status classification, tobacco use history, and indication for THA (primary osteoarthritis vs. other). Overlap weights derived from the propensity score were used in the Cox proportional hazards regression models to estimate weighted HRs for each complication. Overlap weighting was chosen because it balances covariates by up-weighting patients in the region of clinical equipoise and down-weighting those who would almost certainly receive one approach regardless of other factors. Covariate balance after weighting was assessed using standardized mean differences (SMD). After applying the overlap weights, the between-group SMDs of the variables used to create the propensity score model were all less than the accepted threshold of 0.1, indicating excellent balance. Among patients who experienced complications, the type, severity, need for surgical intervention, and resolution of the issue were compared between groups using chi-square tests. Post hoc power calculations for each complication type are provided in Supplementary Table 2. All statistical tests were two-sided, and p-values less than 0.05 were considered statistically significant. All analyses were conducted using SAS version 9.4M8 (SAS Institute, Inc.) and R version 4.4.1 (R Foundation for Statistical Computing).

## Results

A total of 618 complications occurred: 201 among 4,451 hips in the DAA group (4.5%) and 417 among 7,332 hips in the PA group (5.7%) (Table II). Unweighted survivorship free of any complication at 1 year was higher in the DAA cohort (95.9% vs. 94.2%; Fig. 1-A), but after weighting, overall complication risk did not differ between approaches (hazard ratio [HR] 0.89; 95% confidence interval [CI], 0.75-1.06; p = 0.208).

**TABLE II:** Distribution of Complication Types by Surgical Approach

Complication Type	DAA (n = 4,451, %)	PA (n = 7,332, %)	Total (n = 11,783, %)
Any complication	201 (4.5)	417 (5.7)	618 (5.2)
Dislocation	15 (0.3)	139 (1.9)	154 (1.3)
Superficial infection	68 (1.5)	54 (0.7)	122 (1.0)
Deep infection	20 (0.5)	52 (0.7)	72 (0.6)
Periprosthetic fracture	82 (1.8)	158 (2.2)	240 (2.0)
Aseptic loosening	4 (0.09)	4 (0.05)	8 (0.07)
Neurologic deficit	12 (0.3)	10 (0.1)	22 (0.2)

DAA = direct anterior approach, and PA = posterior approach.

Kaplan–Meier curves showing survivorship free of complication within 1 year following THA, stratified by surgical approach.

**Fig. 1-A:**
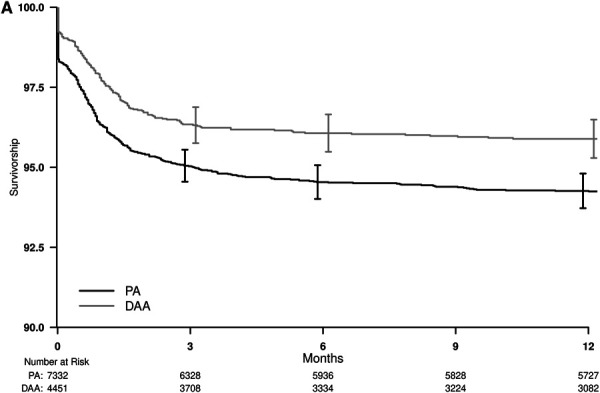
Survivorship free of any complication.

**Fig. 1-B:**
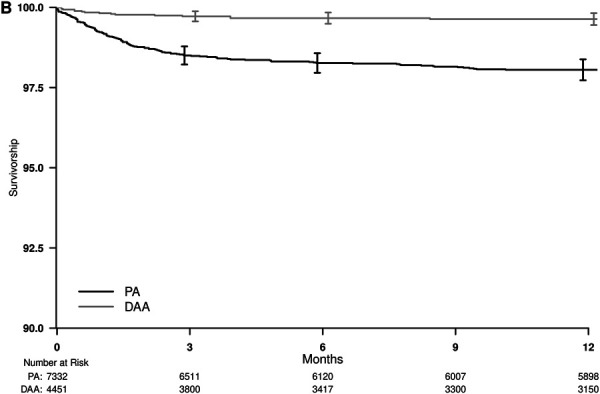
Survivorship free of dislocation.

**Fig. 1-C:**
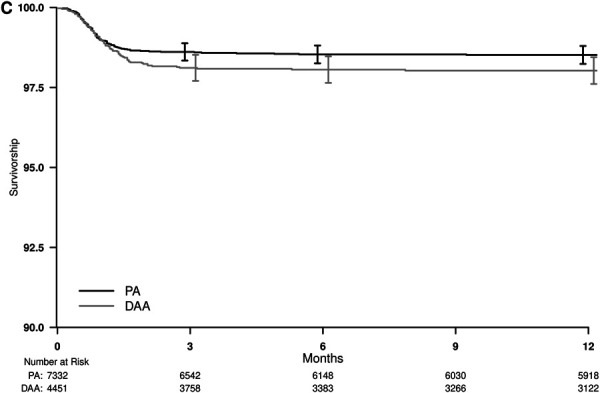
Survivorship free of any infection. DAA = direct anterior approach, PA = posterior approach, and THA = total hip arthroplasty

Among patients who experienced a complication, the need for additional surgical intervention was similar between groups (41.3% for DAA vs. 34.8% for PA; p = 0.1155) (Table III). When compared across all Clavien-Dindo grades, complication severity distribution differed between approaches (p = 0.008), primarily driven by a higher proportion of Grade II complications in the DAA cohort and higher proportions of Grade I and IV complications in the PA cohort (Supplementary Table 1). However, after weighting, the odds of experiencing a severe complication (Grade III to V) did not differ significantly between approaches (adjusted odds ratio [OR], 1.34 for PA compared with DAA; 95% CI, 0.95-1.91; p = 0.099). Overall complication resolution was similar between approaches (86.6% for DAA vs. 84.4% for PA; p = 0.4808) (Table IV).

**TABLE III:** Surgical Interventions for the Complications by Approach

	Complications		
	Direct Anterior(N = 201)	Posterior (N = 417)	p
Required surgery?, n (%)			0.1155*
No	118 (58.7)	272 (65.2)	
Yes	83 (41.3)	145 (34.8)	
Total no. of operations			0.1951†
N	83	145	
Mean (SD)	1.2 (0.48)	1.4 (1.02)	
Median	1.0	1.0	
Range	1.0, 3.0	1.0, 8.0	
Total no. of operations, n (%)			0.6865*
1	70 (84.3)	113 (77.9)	
2	10 (12.0)	19 (13.1)	
3	3 (3.6%)	7 (4.8)	
4	0 (0.0)	3 (2.1)	
5	0 (0.0)	1 (0.7)	
7	0 (0.0)	1 (0.7)	
8	0 (0.0)	1 (0.7)	
NA (did not require surgery)	118	272	
Clavien-Dindo, n (%)			0.0076*
I	31 (15.4)	88 (21.1)	
II	74 (36.8)	96 (23.0)	
III	53 (26.4)	122 (29.3)	
IV	43 (21.4)	110 (26.4)	
V	0 (0.0)	1 (0.2)	
Clavien-Dindo, n (%)			0.0582*
I/II	105 (52.2)	184 (44.1)	
III/IV/V	96 (47.8)	233 (55.9)	

* χ^2^ p-value.

† Wilcoxon rank sum p-value.

**TABLE IV:** Resolution of Complications by Type and Surgical Approach

Complication Type	DAA (Resolved/Total, %)	PA (Resolved/Total, %)	p
Dislocation	15/15 (100)	138/139 (99.3)	0.999*
Superficial infection	67/68 (98.5)	54/54 (100)	0.3709†
Deep infection	10/20 (50.0)	17/52 (32.7)	0.0442†
Fracture	75/82 (91.5)	141/158 (89.2)	0.5862†
Loosening	4/4 (100)	4/4 (100)	-
Neurologic deficit	6/12 (50.0)	3/10 (30.0)	0.4149*

DAA = direct anterior approach, and PA = posterior approach.

* Fisher exact p-value.

† χ^2^ p-value.

The total number of subsequent procedures related to complications was 83 in the DAA cohort and 145 in the PA cohort, though the mean number of procedures per complication was identical between approaches (0.49 ± 0.66 for DAA vs 0.49 ± 0.90 for PA; p = 0.9588), and the distribution of procedures per complication did not differ (p = 0.4694).

Survivorship free of dislocation at 1 year was higher in the DAA cohort (99.6% vs 98.1%; Fig. 1-B), and weighted analyses demonstrated a significantly higher risk of dislocation in the PA cohort compared with the DAA cohort (HR, 5.20 for PA compared with DAA; 95% CI, 3.04-8.90; p < 0.001). Most dislocations were managed initially with closed reduction, and resolution rates were high in both groups (Table IV).

Survivorship free of any infection at 1 year was slightly higher in the PA cohort compared with the DAA cohort (98.5% vs. 98.0%; Fig. 1-C), and weighted analyses demonstrated a higher risk of any infection in the DAA group (HR, 1.71; 95% CI, 1.27-2.29; p < 0.001). This difference was driven by superficial infection, which was more common in the DAA cohort (HR, 2.43; 95% CI, 1.68-3.53; p < 0.001), whereas deep infection risk did not differ significantly between approaches (HR, 0.85; 95% CI, 0.50-1.47; p = 0.565).

Periprosthetic fracture was the most common complication overall (1.8% DAA vs. 2.2% PA) and did not differ significantly after weighting (HR 0.94; 95% CI, 0.71-1.25; p = 0.692). Of the 240 fractures, 141 (58.8%) occurred intraoperatively and 99 (41.2%) postoperatively. Intraoperative fractures occurred more frequently in the PA cohort (1.5% vs. 0.7%; OR 2.3; 95% CI, 1.5-3.4; p < 0.001). Fracture resolution was similar between approaches (Table IV). Aseptic loosening was rare (8 total events) and did not differ significantly between approaches (HR, 1.99; 95% CI, 0.47-8.45; p = 0.351). Neurologic deficit was uncommon and did not differ significantly between approaches (HR, 2.08; 95% CI, 0.88-4.93; p = 0.097); most neurologic deficits in the DAA cohort were isolated sensory deficits, whereas those in the PA cohort more often involved motor or combined motor-sensory deficits.

## Discussion

Differences in complication profiles between the DAA and PA for THA have been described previously^1,2^. However, the severity, early clinical impact, and resolution of these complications have not been comprehensively studied. In this large, multicenter consecutive series of contemporary THA we found that, although the types of complications differed between DAA and PA THA, the overall complication burden, considering incidence, severity, need for additional interventions, and resolution, was similar between approaches. These findings suggest that neither approach is inherently superior from an overall complication standpoint, and that differences in complication profiles may inform shared decision making between surgeons and patients when weighing the relative risks of specific complications for individual patients.

Although baseline cohorts appeared broadly similar, important differences in variables such as BMI and indication for surgery remained, and propensity score overlap weighting was used to balance measured covariates. Although the PA cohort underwent more total procedures, this reflected the greater overall number of complications; per-complication intervention burden and resolution were similar between approaches.

Consistent with most prior studies, dislocation occurred significantly more often after the PA vs. the DAA. Large registry data from the Kaiser Permanente (>38,000 THAs)^4^ and the Australian Orthopaedic Association (>120,000 THAs)^16^ have similarly reported a higher risk of dislocation or revision for instability with the PA. Future work comparing contemporary variations of both approaches, particularly those involving soft-tissue preservation, may clarify whether specific technical refinements alter complication profiles^17-19^.

The higher infection risk with the DAA was driven by superficial infections, consistent with prior reports of increased wound issues with this approach^11,20^. Wilson et al. demonstrated that superficial wound infections after the DAA, when appropriately managed, did not lead to an increased risk of deep infection^10^. When deep infection occurred, a larger percentage of patients in the PA group were on chronic antibiotic suppression at last follow-up and considered not resolved compared with the DAA group. Although uncertain, there may be differences in periprosthetic joint infection management practices between surgeons, centers, or in patient-specific infection risk profiles that could account for these differences.

In contrast to some prior reports suggesting a higher risk of aseptic loosening and neurologic injuries with DAA^9,16^, we did not observe significant differences after propensity score adjustment. The distinct pattern of neurologic deficits between approaches likely reflects the different neural structures at risk during each exposure. Periprosthetic fracture was the most common complication in both groups and occurred at comparable rates (1.8% DAA vs. 2.2% PA; HR 0.94; p = 0.692). Notably, intraoperative fractures accounted for nearly 60% of all periprosthetic fractures and were significantly more frequent with the PA vs. DAA (1.5% vs. 0.7%; OR 2.3; p < 0.001), contrasting with prior reports of elevated intraoperative fracture risk with the DAA during the learning curve. Higher rates of cemented femoral fixation in the PA cohort (11.3% vs. 2.7%) may reflect differences in patient anatomy and implant strategy not fully captured by measured covariates. Ultimately, fracture risk is multifactorial, with patient factors (bone quality, age, BMI, sex) and implant-related factors (stem design, fixation method) contributing independently of approach^2,8^.

This study has limitations. As a retrospective analysis, it is subject to inherent selection bias, which we attempted to mitigate through propensity score weighting. While major events requiring reoperation, closed reduction, or revision were reliably captured across centers, registry-based and chart-based identification may have undercaptured minor complications. In particular, identification of neurologic deficits relied on registry coding and clinical documentation, which likely underestimates transient sensory symptoms such as lateral femoral cutaneous neuropraxia that are often captured only through dedicated patient-reported screening. The high resolution rates observed for dislocation and aseptic loosening also partly reflect how these end points were defined and do not directly index the morbidity of the initial event. Surgeon-specific factors such as operative volume and evolving experience were not modeled, as this would require time-dependent analyses beyond the scope of this study. The use of robotic or navigation-assisted technology was not recorded in the data set and could not be evaluated. Finally, the Clavien-Dindo classification may group events with differing clinical burden within the same grade; we addressed this by also reporting the number of subsequent procedures required per complication.

In conclusion, although the type of surgical complication differed between DAA and PA THA, the overall severity, early clinical impact, and resolution of complications appeared similar. These findings suggest that the choice of surgical approach can be guided primarily by patient-specific factors, with knowledge of these differences in complication profiles informing shared decision making between surgeons and patients.

## Appendix

Supporting material provided by the authors is posted with the online version of this article as a data supplement at jbjs.org (http://links.lww.com/JBJSOA/B277). This content was not copyedited or verified by JBJS.
